# Closing the Loop: Exploring Student-Mentors’ Dual Roles in a Longitudinal Group Mentorship Course

**DOI:** 10.5334/pme.1824

**Published:** 2025-12-04

**Authors:** Allison Odger, Yvonne Steinert

**Affiliations:** 1 Institute of Health Sciences Education, McGill University, Montreal, Quebec, Canada; 2 Faculty of Medicine and Health Sciences, McGill University, Montreal, Quebec, Canada

## Abstract

**Purpose:**

Peer mentorship has emerged as an important strategy for supporting medical students, demonstrating multiple benefits for mentees and mentors. However, to our knowledge, no studies have focused on medical students’ simultaneous experiences as students (receiving mentorship) and near-peer mentors (providing mentorship). This study explored students’ experiences as students and mentors in a longitudinal, group mentorship course.

**Method:**

The authors used interpretive description to explore how senior medical students acting as near-peer mentors understood their dual roles. In-depth semi-structured interviews were conducted between April and October 2023. Additionally, students were asked to record audio diaries. Using an iterative, reflexive approach, the authors conducted a constant comparative data analysis.

**Results:**

20 students completed 2 interviews and audio diaries. Four themes were identified: Participants understood their dual roles to be complementary, highlighting a continuity between being students and near-peer mentors, which they called “closing the loop.” Many participants cited their experiences in the course as the reason they decided to become student mentors, expressing their desire to “pay it forward.” Mentorship roles reminded them of their experiences as new medical students, and “gazing backwards” helped boost their confidence and renew their empathy. Several participants drew attention to how their mentorship role helped them “imagine the future,” reaffirming their desire to continue mentoring or teaching.

**Discussion:**

Being in two roles was perceived as a positive experience. Dual roles helped boost students’ confidence and empathy while fostering meaningful reflection on career trajectories, findings that could help inform or enhance peer mentorship programs.

## Introduction

Mentorship has been recognized as a powerful strategy for supporting medical students on their path to becoming physicians [1]. During their education, students encounter many stressful situations [2,3], and mentorship programs can help support students’ personal and professional development [4,5,6]. Near-peer mentorship builds on the strengths of mentorship programs and offers a way to address the increasing demand for mentors while enhancing students’ learning experiences; such programs can also be beneficial for students as learners and mentors, as they can provide academic, social, and emotional support for mentees and can reinforce medical knowledge and teaching skills for near-peer mentors [7,8,9].

Near-peer mentorship also falls within the student-as-teacher literature, which primarily focuses on fostering students’ teaching skills [10,11,12,13]. Research in this area has described programs that aim to prepare students for their roles as teachers, analyzing course content (what they teach), modalities (how they teach), assessment strategies (evaluating how well they teach), and outcomes (gauging the impact of their teaching) [10]. The near-peer mentorship and student-as-teacher literature have both highlighted the effectiveness of peer-based models, recommending formal and longitudinal programs. However, to our knowledge, no studies have focused on medical students’ simultaneous experiences as students (receiving mentorship) and near-peer mentors (providing mentorship).

While researchers have examined the dual roles of physicians as health care providers and patients [14], similar attention has not been paid to medical students’ dual roles within a near-peer mentorship program, and no studies have examined whether holding such roles enriches or detracts from students’ experiences, To address this gap, we sought to understand the experiences of student-mentors in a group mentorship course and asked the following research questions: (1) *How did medical students understand and navigate their dual roles as students and near-peer mentors in a longitudinal group mentorship course?* (2) *In what ways did these dual roles influence their educational trajectories?* We anticipated that our study would contribute to the near-peer mentorship literature and to educational practice in two ways. First, the insights gained could help optimize future near-peer mentorship programs by identifying areas that can help to improve student experience. Second, if participating in dual roles is found to be beneficial, this model could be adopted and supported in other medical education contexts.

Our study design and analysis were informed by the work of Goffman [15]. Using a theatre metaphor, Goffman understood everyday life as a stage where people take on various roles with various expectations, and within this context he theorized how social norms shape individuals while individuals, in turn, shape social norms [16 (p288)]. From Goffman’s perspective [15], roles are dynamic and created in face-to-face interactions, and his work can be used to analyze how people define themselves and how they are understood by others [16 (p278)]. In health sciences education, scholars have used Goffman’s role theory to analyze informal learning in clinical settings [17], interprofessional teamwork [18], and students’ clinical experiences [19]. Additionally, Goffman’s contributions to role theory provide a useful framework for understanding if, when, and how roles interact, and whether there is tension or conflict. In our study, we used Goffman’s conceptualization of roles to understand how participants negotiated being students and near-peer mentors, how they made meaning of these experiences, and whether these simultaneous roles helped or hindered them along their paths to becoming physicians.

## Study Design and Methodology

We used interpretive description (ID) as our study design [20]. ID embraces the subjectivity and contextual nature of human experiences while maintaining rigour through iterative, reflexive, and concurrent analysis [21]. It is a pragmatic, flexible methodology that looks for patterns, diversity, and contradictions, with the goal of translating participants’ lived experiences into practical outcomes [20,21].

We conducted semi-structured interviews and solicited audio diaries to attend to participants’ understandings of their dual roles and capture their reflections on those experiences. We acknowledged our role in data generation and engaged in reflexivity throughout all research phases. The first author, a medical anthropologist with a PhD in Social Anthropology and experience conducting ethnographic research, was a Postdoctoral Fellow and relatively new to health sciences education as a discipline. Also, she was not involved in the planning or coordination of the mentorship course. The second author is a clinical psychologist. She brings her research experience, including faculty development, professionalism, and identity formation, to this study. She is also a member of the course coordinating committee, which oversees this mentorship course. To address any potential power dynamics and avoid participants feeling pressured to perform in any way, the first author was responsible for recruiting participants and conducting interviews. Additionally, the study design and data analysis benefited from the authors’ diverse positionalities, which fostered questioning and reflection on assumptions. YS had “insider” knowledge about the mentorship course and AO had an “outsider” perspective. In ID, the researcher’s background, knowledge, and experiences are not seen as bias to be removed, but rather as valuable to providing insight [21].

## Research Context

Undergraduate medical education (UGME) in Canada requires 3–4 years of study. The curriculum begins with foundational science courses, followed by a multi-year clinical rotation (known as a “clerkship”). At the institution where we conducted our study, students are expected to complete coursework in the fundamentals of medicine and dentistry before transitioning to clerkship; the latter is a combination of mandatory core rotations and electives. Another key component of students’ UGME experience is a mandatory four-year longitudinal group mentorship course, Physician Apprenticeship [22,23]. Established in 2005, the goal of the course is to assist medical students in their transition from layperson to physician and to provide a safe and supportive environment to discuss concerns arising from educational experiences. During the course, a physician mentor (an Osler Fellow), 6 junior medical students, and 2 senior medical students who act as near-peer mentors (co-leaders) meet regularly over all 4 years of medical school, beginning with 6 meetings in the first year, 4 meetings in the second, 4 meetings in the third, and 2 meetings in the final year. While these are the minimum number of meetings that students must attend, all groups have the flexibility to meet more frequently. During these group encounters, students can discuss key topics (some predetermined, some generated spontaneously), ask questions, reflect on clinical experiences, and participate in course activities and experiential learning. For example, mandatory activities can include attending a clinical encounter arranged by the Osler Fellow or meeting with an interprofessional team to discuss the importance of interprofessional care. Co-leaders participate in two different groups: as senior medical students in their own student groups, and as near-peer mentors working with more junior medical students.

## Participants and Recruitment

Eligible participants included all third- and fourth-year medical students who held the co-leader position in the spring of 2023 (n = 115). Based on a recruitment strategy developed in collaboration with the course coordinating committee, the first author sent a personalized email to all co-leaders outlining the study purpose, participation requirements, and compensation. In the recruitment email, co-leaders who were interested in participating were asked to respond to AO to schedule an interview.

## Data Collection

We conducted semi-structured interviews to understand participants’ perceptions and lived experiences. During the first interview, the first author (AO) developed rapport with participants and asked them to reflect on their course experiences (see Appendix A). We also solicited audio diaries [24] to capture students’ reflective thoughts about their experiences [25]. To guide audio diary entries, students were asked to reflect on a recent memorable experience in each role, in a 30-second to 5-minute recording (see Appendix B). AO transcribed participants’ audio diaries and drafted additional questions specific to their content for the second interview (see Appendix C), which was designed to generate rich descriptions of how participants understood, navigated, and experienced their dual roles.

AO held all interviews virtually via Zoom to accommodate participants’ busy schedules. Interviews ranged in length from 35 to 69 minutes and were transcribed verbatim. Additionally, AO completed post-interview reflective fieldnotes, summarizing key perspectives and stories, as well as preliminary analytic thoughts.

## Data Analysis

In line with ID [21 (p340)], we conducted constant comparative analysis. This involved immersing ourselves in the data as a whole, creating preliminary themes, extracting data from participant transcripts (interview and audio diary), and holding multiple discussions between the authors. We analyzed all data through open coding followed by focused coding using an iterative-inductive process [26,27]. In June 2023, we started a more intensive analytic process and refined our analysis by probing for new insights and divergent themes. With the extracted data, we created documents for each participant, and highlighted key quotes, how participant narratives aligned with our themes, and posed further questions for reflection. We compared, contrasted, and returned to our themes, narratives, and questions throughout the process to deepen our understanding. Additionally, the first author completed a content analysis of the audio diaries.

We acknowledged our role in the creation of themes, engaging in multiple analytic conversations to think through our assumptions, discuss how our interpretations differed, and deepen our analysis.

## Ethical Considerations

The Institutional Review Board (IRB) approved all study materials. We obtained informed consent from all participants and maintained confidentiality. We labelled notes, recordings, and transcripts by date and participants’ pseudonyms. All names utilized in this article reflect those pseudonyms. Since participants shared personal stories, we took care to ensure they were not identifiable in our research findings; for example, direct quotes capture their perspectives and do not include specific details that could identify the students or the group they attended in the course. To provide an incentive and show appreciation for students’ time/expertise, we offered participants an Amazon or bookstore gift card worth $75.00.

## Research Findings

Out of 115 eligible co-leaders, 22 responded to our recruitment email between April and October 2023; this included 10 third-year and 12 fourth-year medical students (with an average age of 25.5 and 26.3 respectively). Our research findings are based on the 20 participants who completed all aspects of data generation; two participants completed the first interview but did not respond to follow-up emails. We spoke with co-leaders who came from different backgrounds; some participants completed degrees before entering medical school; others had transitioned through a qualifying year after completing their college studies. At the time of our study, fourth-year students had two years of experience as co-leaders while third-year students had only one year. Participants observed that the amount of time they spent in their co-leader role could affect how they understood their dual roles, though all felt confident in their ability to share their perceptions and experiences as students and co-leaders in their groups.

In analyzing the data, we identified four themes in response to our research questions, addressing how participants understood and navigated their dual roles, and how these roles helped shape their educational trajectories: closing the loop, paying it forward, gazing backwards, and imagining the future.

### Closing the Loop

Many participants understood their dual roles in the course to be complementary and interconnected. Most students felt that they could draw on their experiences in one role and apply them to the other, and they described an “overlap” between their roles as mentees and mentors (“like a Venn diagram”). As Bonnie explained:

*…at the end of the day, it’s the same concept, and at the end of the day, I’m still a student in both groups, and I still have questions, and I still get help from other students, so I think there’s definitely a lot of overlap… and I think that overlap is nice ‘cause I think I can bring things that were said in my previous group into my new group*.

A few participants spoke about their roles as more “distinct” (e.g., teaching vs. learning, providing information vs. receiving information); however, the majority described a circularity – or continuity – between their two roles. Goffman describes the consequences of taking on multiple roles, which can result in strain, ambiguity, or frustration [15]. However, we saw the opposite in participants’ narratives, highlighting the complementarity of the dual roles. Emily described the circular nature of holding two roles as “closing a mentorship loop.” Joan described the continuity as follows:

*It’s like a spectrum, and, you know, you’re just at two different points of the same spectrum, and the fact that I’ve been through my own course, helps me be able to be a good support ‘cause then I know what I thought was helpful during discussions with the med students*.

As co-leaders, participants were in a unique position because they had gone through the same process as their students only 1–2 years earlier. This “natural continuity” between dual roles was also highlighted in the narratives of participants who did not have an ideal group experience as students. Ultimately, they could still imagine the ways in which their dual roles could relate and how they could simultaneously draw upon past and current experiences.

Finally, participants were generally enthusiastic about their time as near-peer mentors and student-mentees. Although we probed for negative experiences, specifically asking whether there was any tension between their dual roles, they did not describe any difficulties. Similarly, we gave participants the opportunity to discuss dilemmas in their audio diaries, but these stories were limited to medical school and certain Osler Fellows; no dilemmas were described in terms of their dual roles.

### Paying it Forward

For many participants, their experiences as students in the course informed their decision to become co-leaders. Despite a passion for, and at times previous history of, mentorship, several participants identified the role of being a student in the course as a motivating factor. Participants’ narratives also highlighted how their student experience not only shaped their choice to become a co-leader but also influenced how they approached this role. In Bonnie’s words:

*I had [a] really good experience with my group, so I wanted to be able to give back to other students as well, and so that was my main motivation… I had a good experience so I wanted new incoming students to have a good experience too, and I felt like I could measure up to the task*.

Participants discussed their desire to support junior students in the same way their mentors had done for them. In essence, they wanted to “give back” to the next cohort. Beginning medical school can be a fraught time, and having the support of co-leaders helped navigate the norms and expectations of this new cultural context. Joan’s narrative highlighted how her group shaped her desire to “recreate” a supportive experience for another cohort of students:

*…as someone who came from, like, a family where nobody’s a doctor, and [I] didn’t really know what I was getting into in medical school, having an older student being able to tell me, ‘Okay, this is what you anticipate; this is what’s gonna happen’, made the transition that much easier, and made me kind of be able to know what I was getting into. So, I wanted to be able to give that to younger students as well*.

While most participants wanted to take on the co-leader role because they had an excellent mentee experience, in which they felt heard and supported by their Fellow and group members, other students discussed less positive experiences. Cheryl did not have an ideal experience with her Fellow but wanted to become a co-leader to “make sure [another group of students] had a good experience” and did not have the same experience as her. Ultimately, participants’ own group experiences shaped their choices to take on the co-leader role, as they wanted to ensure that students received meaningful support.

### Gazing Backwards

By listening to their students’ concerns and answering practical questions, co-leaders were often reminded of their own experiences as junior medical students. From their perspective, they had changed significantly between first and third/fourth year, and reflecting on these changes had two effects.

By observing their growth, students described feeling more confident in their knowledge and skills. For example, Sabrina reflected in her audio diary on a time when she stepped in for her Fellow at the simulation centre:

*It was quite cool because I had done the same activity myself not too long ago and so, it was nice to see that just a few years later I was able to, you know, shift roles from that of a student to that of a supervisor*.

Despite only being a couple of years ahead, Sabrina felt her students valued her perspective. It was a formative moment that demonstrated to her how far she had come in her educational trajectory.

Co-leaders’ relationships with their students worked to “remind” them be more empathetic towards junior learners. Participants described how they were often told they would lose their empathy in medical school, and some spoke about how this was already happening. Margaret’s experience in clerkship led to frustration with more junior students who were new to the clinical space. However, she saw her co-leader group as providing a “grounding experience,” which shaped her approach to working with more junior learners generally, saying:

*I think the first-year group sort of allows me to be reminded of, like, we were all there; we were all puppies, like, at some point, and sort of clueless, and had to be shown where things were, and I think there’s a parallel between that and…not being judgemental. I think it carries into the same with interacting with other students., Sort of offering the same kindness, empathy, patience, non-judgemental approach*.

Participants’ narratives also highlighted how their junior students sometimes became “mirrors” for them, where they could see their former selves, including their past worries, concerns, and curiosity. For some, this new vantage point served to re-energize them, with their students’ excitement helping them to remember why they chose to become physicians, as noted in Ronald’s co-leader audio diary:

*…it was really nice to kind of hear how excited they were for the future, how much they were looking forward towards clerkship, and even though I was at the end of my third year getting a bit tired, a bit burnt out, hearing from them about how much they’re still looking forward to, how excited they are, just reminded me of why I got into this in the first place. And how excited I was when I was in their shoes…*

Working as co-leaders gave participants the opportunity to benefit from multiple perspectives. In addition, there was no conflict between the participants’ two roles; negotiating them simultaneously meant they could inform one another in a way that was personally meaningful to participants. According to Goffman, roles are relational and interdependent, made in social interactions [15]. By “gazing backwards,” participants’ dual roles worked together, resulting in complementarity instead of conflict.

### Imagining the Future

Participants reported that their co-leader roles either re-affirmed or inspired a desire to teach and mentor in the future. In Brandon’s co-leader audio diary, he described his relationship with his students as both important and fulfilling:

*…in terms of becoming a physician in the future, it really solidified my passion and desire to continue to have that mentor-mentee role, as a mentor in the future with future medical students, residents, other staff, once I’m much, much older. And it proved to me that this role means something, and this role can have a large impact*.

For Roland, his experience as a co-leader worked to shape his educational trajectory and how he imagined his future as a resident and beyond, as witnessed in his audio diary:

*I already knew the importance of mentoring, but seeing the difference it makes in my mentees meant the world to me. And [it] even somehow shifted my plans about my [future] workplace. Indeed, at first, I wanted to work in a community centre, but now being a co-leader and mentoring those aspiring medical students, it makes me lean more towards working in an academic centre when I become a staff physician. So, to keep teaching the future generation and being a great and good mentor…*

Participants also shared how their co-leader role offered valuable lessons that worked to shape their understanding of the kind of mentors or educators they wanted to become. Jimmy learned the importance of “not making assumptions” and “taking the time to understand others” before voicing his own perspectives. Bonnie reflected on a co-leader meeting where she shared a clerkship “failure,” and came away from that experience with the understanding that talking about her mistakes and sharing her struggles could be beneficial. In fact, she wanted to continue working on this approach with students, stating: “…I think it’s just important in the future for me to remember to try to be a little bit more honest about the challenges and mistakes that we face so that we can help other people.”

In different ways, participants’ narratives demonstrated how the co-leader role worked to shape their imaginings of what kind of mentor-educator they could become in the future. According to Goffman, *distancing* may occur when a person does not identify with their role [15]. However, participants’ narratives demonstrate the impact of *embracing* a role. With role embracement, the role can become a part of who they are, rather than just a performance, which can translate into increased commitment or dedication to the role. As near-peer mentors who are also student-mentees, the co-leader role reaffirmed students’ desire to continue teaching and/or mentoring in the future. In this sense, these roles became part of them, rooted in their face-to-face interactions with their students and shaping who they were throughout their medical education trajectory.

## Discussion

Participants understood their dual roles to be complementary and interconnected. Participants wanted to play a mentorship role in the course and “pay it forward” to another cohort of students. By meeting with their co-leader group, participants were reminded of what it was like to be a new medical student and why they chose to go into medicine in the first place. These reminders had two main effects: they helped to build students’ self-confidence and shape their commitment to being more empathetic to junior learners. Their co-leader role as near-peer mentors, also worked to reaffirm or inspire their desire to mentor or teach in the future.

Recent studies have demonstrated the positive effects of near-peer programs at the undergraduate level. For example, one study demonstrated increased exam preparedness and confidence of senior medical students by pairing them with residents [28]. Another highlighted the benefits of having clinical students provide emotional support for pre-clinical students [29]. A third study illustrated the positive impact of near-peer mentors helping incoming students from underrepresented groups transition into medical school [8]. While studies have highlighted the benefits of such programs, explorations of lived experiences in near-peer mentorship in medical education remains sparse.

Facilitating reflective discussions has been identified as an “optimal organizational feature” in group-based mentorship courses [1 (p277)]; in this study, holding dual roles helped foster deep and meaningful reflection. The longitudinal nature of the course and the development of relationships in each of these roles also provided a space for participants to slow down, reflect on their experiences, and consider their future as mentors and educators. This finding aligns with previous scholarship that asserts that near-peer mentorship can “cultivate” interest in teaching and mentorship [24].

Medical education includes multiple transition points, which can pose many challenges for students [2]. Based on participants’ narratives, we concluded that navigating their co-leader role served to help build their confidence during a period when they were experiencing numerous stressors as clerks. Additionally, new medical students are often warned that they are at risk of losing their empathy as they progress through their training [30]; working with junior medical students as near-peer mentors helped renew senior students’ commitment to empathy and reenergize them on their path to becoming physicians.

In analyzing our data and developing our themes, we were able to draw on the work of Goffman to attend to the nuances of participants’ roles as medical students and co-leaders. A symbolic interactionist approach to roles, as described by Goffman, embraces and emphasizes the social context in which such roles exist as well as the subjectivity of participants’ experiences. Goffman argued that roles are “not gliding surfaces that conceal the true person;” instead, they are dynamically shifting and become a part of people [16 (p290)]. Participants’ narratives highlighted how they understood being a mentee and a near-peer mentor at the same time, and how these roles were not something they put on and took off; these roles were a part of their everyday lives as students. Goffman’s broad conceptualization of roles also served to deepen our interpretive lens. By thinking of roles as dynamic, we could attend to how participants’ understanding of their roles evolved over time; for example, we could explore how the knowledge and skills gained in their co-leader role could be applied in their student role and in their future as physicians. Goffman argued that roles are created in face-to-face interactions; this was useful for exploring participants’ reflections on how they negotiated their roles with others and the impact those roles had on them. Finally, Goffman’s work provided insight into the concepts of role *distancing* and role *embracement*. Role distancing can be done “in an effort to signal to the surroundings that they are in fact something more than what the social role would seem to imply [31 (p108)].” In our study, participants’ embracement of, and identification with, their dual roles, had a positive impact on their path to becoming physicians and possibly future mentors.

Our research findings showed that, even though students simultaneously held two roles with different expectations, they did not experience any dissonance or tension between them. Participants’ reflections repeatedly highlighted the continuity and interconnectedness of being a student and near-peer mentor at the same time, and how the complementarity of these roles was productive. Goffman helps us to think about roles on two levels: how the individual engaged with the role (distance, tension, immersion, embracing) and how the role impacted them (or became a part of them). Importantly, our approach explored how negotiating these roles simultaneously enabled them to inform one another in ways that were personally meaningful to participants. Participants’ narratives highlighted several benefits of holding dual roles, including opportunities to reinforce their learning and renew their commitment to empathy toward junior learners. This study suggests that providing resources and support for students to take on dual roles can create space for deeper reflection and richer insights. While near-peer models, including the student-as-teacher role, have been explored for their benefits and impact, little to no attention has been paid to the concept of dual roles. Our study enriches this literature by focusing on the benefits of students holding dual roles, and we hope these insights will contribute to a relatively small area of the literature that seeks to understand (or value) dual roles and how they can be optimized in educational practice.

## Strengths and Limitations

Of the 22 co-leaders we recruited, 20 completed all aspects of participation. We considered participants’ involvement a strength of our study. They took the time to participate in two interviews and record two audio diary entries, sharing vulnerable and meaningful experiences from their groups. As a qualitative research method, solicited audio diaries generated rich narratives via storytelling, capturing participants’ personal reflections.

At the same time, we recognize that those who responded to our recruitment email may have been particularly keen to voice their perspectives. Numerous factors (including students’ educational backgrounds, life pathways, and time in their co-leader role) shaped participants’ experiences. Additionally, the COVID-19 pandemic and subsequent teaching online played a role in how students understood and navigated PA. Therefore, our study findings reflect the specific experiences of our participants and are not representative of all co-leaders nor near-peer mentors in other settings.

## Implications for Practice and Research

Despite these limitations, our study has practical implications for medical education. Based on our findings, holding two roles can help boost students’ confidence, foster reflection, promote empathy, offer moments of deep satisfaction, and provide opportunities to reinforce learning and explore new ideas. Educators may wish to consider these benefits when designing teaching and learning opportunities for their students. The group mentorship model described here could also serve as a stimulus for developing new, or enhancing existing, mentorship programs in medical education. For example, mentorship programs could incorporate opportunities for students to take on dual roles (both receiving and providing mentorship) to foster their confidence, reflection, empathy, and learning. Moreover, if dual roles are promoted, educators may wish to strengthen their inclusion. Offering mandatory training workshops could help students prepare for their dual roles. Additionally, creating a formal space for students to debrief on their experiences and share reflections would be worthwhile. From a research perspective, medical educators have conducted program evaluations of near-peer mentorship programs using both quantitative and qualitative methods. However, further research is needed to better understand students’ lived experiences in near-peer mentorship programs. Qualitative research methods (e.g., participant observation, in-depth interviews, audio diaries) would be ideal for capturing the perspectives of near-peer mentors and mentees. Future studies could also explore the influence of participating in mentorship roles by asking: how do students carry their mentorship experiences into their postgraduate training and beyond? And how does holding dual roles influence students’ professional identities?

## Conclusion

To our knowledge, this is the first study to explore the perceptions and experiences of participants who are both students and near-peer mentors within the same mentorship course. Our results illustrated how these two roles operated in complementary ways, with past and present experiences as medical students shaping students’ roles as co-leaders and vice versa. Additionally, we found that working with junior students in a mentorship setting could have a positive impact on more senior students, helping to build their confidence and reminding them of their commitment to empathy. Finally, our findings suggest that this dual role and the relationships that resulted from being both a near-peer mentor and student fostered deep and meaningful reflections about who students were and who they wanted to become.

## Additional File

The additional file for this article can be found as follows:

10.5334/pme.1824.s1 Appendices. Appendix A to C.

## References

[1] Skjevik EP, Boudreau JD, Ringberg U, Schei E, Stenfors T, Kvernenes M, et al. Group mentorship for undergraduate medical students-a systematic review. Perspect Med Educ. 2020;9(5):272–80. DOI: 10.1007/S40037-020-00610-3 32820416 PMC7550430

[2] Teunissen PW, Westerman M. Opportunity or threat: the ambiguity of the consequences of transitions in medical education. Med Educ. 2011;45(1):51–9. DOI: 10.1111/j.1365-2923.2010.03755.x 21155868

[3] Atherley A, Dolmans D, Hu W, Hegazi I, Alexander S, Teunissen PW. Beyond the struggles: a scoping review on the transition to undergraduate clinical training. Med Educ. 2019;53(6):559–70. DOI: 10.1111/medu.13883 31012141 PMC6593677

[4] Driessen E, Overeem K. Mentoring. In: Walsh K, editor. Oxford Textbook of Medical Education. Oxford: Oxford University Press; 2013. pp. 265–74. DOI: 10.1093/med/9780199652679.003.0023

[5] Burgess A, van Diggele C, Mellis C. Mentorship in health professions: a review. Clin Teach. 2018;15(3):197–202. DOI: 10.1111/tct.12756 29318730

[6] Wu J, Olagunju AT. Mentorship in medical education: reflections on the importance of both unofficial and official mentorship programs. BMC Med Ed. 2024;24:1–3. DOI: 10.1186/s12909-024-06248-7 PMC11523898 39472896

[7] Singh S, Singh N, Dhaliwal U. Near-peer mentoring to complement faculty mentoring of first-year medical students in India. J Educ Eval Health Prof. 2014;11(12). DOI: 10.3352/jeehp.2014.11.12 PMC4309947 24980428

[8] Prunuske A, Houss B, Wirta Kosobuski A. Alignment of roles of near-peer mentors for medical students underrepresented in medicine with medical education competencies: a qualitative study. BMC Med Educ. 2019;19(1):417. DOI: 10.1186/s12909-019-1854-x 31711472 PMC6849195

[9] Abdolalizadeh P, Pourhassan S, Gandomkar R, Heidari F, Sohrabpour AA. Dual peer mentoring program for undergraduate medical students: exploring the perceptions of mentors and mentees. Med J Islam Repub Iran. 2017;31:2. DOI: 10.18869/mjiri.31.2 28638809 PMC5473101

[10] Cohen A, Steinert Y. Teaching medical students to teach: A narrative review and literature-informed recommendations for student-as-teacher curricula. Acad Med. 2022;97(6):909–922. DOI: 10.1097/ACM.0000000000004608 35108235

[11] Shannon K, Talarico S. Preparing the next generation of teachers. In: Steinert Y, editor. Faculty Development in the Health Professions. Second Edition. Singapore: Springer Nature Singapore Pte Ltd; 2025. pp. 405–428. DOI: 10.1007/978-981-97-9372-3_18

[12] Freret T, Rana J, Schwartzstein RM, Gooding HG. Twelve tips for implementation of “student-as-teacher” programs. Med Teach. 2017;39(12):1221–1226. DOI: 10.1080/0142159X.2017.1333591 28598708

[13] Erlich DR, Shaughnessy AF. Student–teacher education programme (STEP) by step: Transforming medical students into competent, confident teachers. Med Teach. 2014;36(4):322–332. DOI: 10.3109/0142159X.2014.887835 24597597

[14] Rowland P, Kuper A. Beyond vulnerability: how the dual role of patient-health care provider can inform health professions education. Adv Health Sci Edu. 2018;23:115–31. DOI: 10.1007/s10459-017-9777-y 28456855

[15] Goffman E. The presentation of self in everyday life. New York: Anchor Books; 1959.

[16] Hacking I. Between Michel Foucault and Erving Goffman: between discourse in the abstract and face-to-face interaction. Economy and Society. 2004;33(3):277–302. DOI: 10.1080/0308514042000225671

[17] Cantillon P, De Grave W, Dornan T. Uncovering the ecology of clinical education: a dramaturgical study of informal learning in clinical teams. Adv Health Sci Educ Theory Pract. 2021;26(2):417–35. DOI: 10.1007/s10459-020-09993-8 32951128 PMC8041675

[18] Lewin S, Reeves S. Enacting ‘team’ and ‘teamwork’: using Goffman’s theory of impression management to illuminate interprofessional practice on hospital wards. Soc Sci Med. 2011;72(10):1595–602. DOI: 10.1016/j.socscimed.2011.03.037 21549467

[19] Vanstone M, Grierson L. Thinking about social power and hierarchy in medical education. Med Educ. 2022;56(1):91–7. DOI: 10.1111/medu.14659 34491582

[20] Thorne S. Interpretive description: Qualitative research for applied practice. New York: Routledge; 2016.

[21] Thompson Burdine J, Thorne S, Sandhu G. Interpretive description: A flexible qualitative methodology for medical education research. Med Educ. 2021;55(3):336–43. DOI: 10.1111/medu.14380 32967042

[22] Steinert Y, Boudreau JD, Boillat M, Slapcoff B, Dawson D, Briggs A, et al. The Osler Fellowship: an apprenticeship for medical educators. Acad Med. 2010;85(7):1242–9. DOI: 10.1097/ACM.0b013e3181da760a 20375831

[23] Boudreau JD, Macdonald ME, Steinert Y. Affirming professional identities through an apprenticeship: insights from a four-year longitudinal case study. Acad Med. 2014;89(7):1038–45. DOI: 10.1097/ACM.0000000000000293 24826856

[24] Monrouxe LV. Solicited audio diaries in longitudinal narrative research: a view from inside. Qualitative Research. 2009;9(1):81–103. DOI: 10.1177/1468794108098032

[25] Cottingham MD, Erickson RJ. Capturing emotion with audio diaries. Qualitative Research. 2020;20(5):549–64. DOI: 10.1177/1468794119885037

[26] Emerson RM, Fretz RI, Shaw LL. Writing ethnographic fieldnotes. Chicago: University of Chicago Press; 2011. DOI: 10.7208/chicago/9780226206868.001.0001

[27] LeCompte MD, Schensul JJ. Analysis and interpretation of ethnographic data: A mixed methods approach. Plymouth: Rowman Altamira; 2012.

[28] Yap AF, Hui Wen, Ruan X, Fong WWS. Development of a personalized near-peer mentoring programme for final-year medical students with residents as mentors. Proceedings of Singapore Healthcare. 2022;31:1–5. DOI: 10.1177/20101058211057325

[29] Lynch TV, Beach IR, Kajtezovic S, Larkin OG, Rosen L. Step Siblings: a Novel Peer-Mentorship Program for Medical Student Wellness During USMLE Step 1 Preparation. Med Sci Educ. 2022;32(4):803–10. DOI: 10.1007/s40670-022-01571-4 35729988 PMC9189789

[30] Seeberger A, Lönn A, Hult H, Weurlander M, Wernerson A. Can empathy be preserved in medical education? Int J of Med Educ. 2020;11:83–9. DOI: 10.5116/ijme.5e83.31cf 32311676 PMC7246122

[31] Jacobsen MH, Kristiansen S. The Social Thought of Erving Goffman. USA: SAGE Publications; 2015. DOI: 10.4135/9781483381725

