# Supplementary material for: Closing the Loop: Exploring Student-Mentors’ Dual Roles in a Longitudinal Group Mentorship Course
Source: Perspect Med Educ. 2025 Dec 4;14(1):905–14. doi: 10.5334/pme.1824 (PMC12679983; doi:10.5334/pme.1824)
Supplement: Appendices. — Appendix A to C. [file pme-14-1-1824-s1.pdf]

Appendix A: Initial Interview Schedule

- What year are you in your program?
- Can you tell me a little bit about yourself?
    - What drew you to medicine and what kind of physician do you hope to be?
        - Why did you decide to become a doctor?
        - Do you have any thoughts as to the speciality of medicine you might be interested in pursuing?
    - Why did you apply to be a co-leader?
- Trajectories
    - As a student, how has the course and its values affected how you think about and experience becoming a physician?
        - What about your understanding of the kind of physician you want to be?
    - As a co-leader, how has the course and its values affected how you think about being and becoming a physician?
        - What about your understanding of the kind of physician you want to be?
        - Can you tell a story to illustrate your point?
    - What are your career aspirations, as you are completing (for 4th years only) medical school?/What are your career aspirations for after you complete medical school? (for 3rd years) Can you tell me a story to illustrate your point?
        - How has the course influenced these aspirations, if at all?
        - If so, can you tell me a story which illustrates the ways in which the course has influenced your aspirations?
- Co-Leader role

- Based on your experiences as a co-leader and a medical student in the course, do you feel as though one of these roles has had a greater impact on your becoming a physician?
    - If so, can you share a story to illustrate your point?
- When you are participating as a co-leader, how does that differ from your participation as a medical student in your own group?
    - Is sitting in one seat more meaningful than another?
- As a co-leader, what have you learned from your experience in the course?
    - How does what you've learned shape your clerkship experiences?
    - Alternatively, how do your clerkship experiences shape what you learn in the course?
- As a Med 3/Med 4 student in your own group, what have you learned from your experience in the course?
    - How does what you've learned shape your clerkship experiences?
    - Alternatively, how do your clerkship experiences shape what you learn in the course?
- How do you find your experiences as a co-leader and as a Med 3/Med 4 student work together?
    - Is there overlap?
    - Do they support each other?
    - Is there a tension between them?

- As a co-leader, how would you describe your relationship with your Fellow?

    - What kinds of tasks/exercises have you been asked to engage in?
    - How are you able to share leadership responsibilities with your Fellow?
    - Can you provide a story to illustrate your point?

- As a student, how would you describe your relationship with your Fellow?
    - Do you see them as a mentor? Do you feel like a mentee?

- Why did you agree to participate in this study? What drew your interest?

Extra Questions:

- How do you see your role as a co-leader?
    - Are there any metaphors which come to mind?
- Do you feel as though you've built relationships/engaged with your group?
- As a co-leader, how do you see your relationship with your students?
    - Do you see yourself as a mentor, a guide, a friend?
- As a co-leader, what do you think you bring to the group?
- What are the advantages and challenges of being a co-leader?
- What have you learned from being a Co-Leader in the course?

- Do you have any questions for me?

Appendix B: Audio Diary Instructions and Prompt

For this exercise, you are being asked to record two separate audio diary entries. Each recording should be at least 30 seconds and no more than 5 minutes. If possible, we would like one entry to be based on your experience as a co-leader and another based on your experience as a Med 3/Med 4 student in your own group.

Prompt: Please tell us a story about something that has recently happened to you during the course. This story should capture an experience, conversation, or dilemma which was meaningful to you and your time in the course as you navigate being a medical student and a co-leader. What impact did this experience, conversation, or dilemmas have on you and how you see yourself in becoming a physician?

Once you have complete both recordings, please send them to the PI.

Do you have any questions for me?

Appendix C: Final Interview Schedule

- Audio Diary Entries
    - General questions
        - Thinking back on your initial response to the described experience/conversation/dilemma, do you have any further reflections?
        - Can you walk me through your thinking in choosing these experiences/conversations/dilemmas in your diary entries?
            - What meaning do these stories hold for you?
            - How did you navigate these situations as a Co-Leader? How did you navigate these situations as a Med 3/Med 4 student?
            - In your co-leader story, did you find your time as a Med 3/Med 4 student helped you navigate/understand the described experience/conversation/dilemma?
            - In your Med 3/Med 4 story, did you find your time as a co-leader helped you navigate/understand the described experience/conversation/dilemma?

Based on the participant's submissions, more specific questions will be tailored to their entries. We also plan to spend part of this final interview asking follow-up questions based on missed opportunities in the initial interview while deepening their previously introduced trajectories.
